# Impact of Educational Program on the Management of Chronic Suppurative Otitis Media among Children

**DOI:** 10.1155/2015/624317

**Published:** 2015-02-22

**Authors:** Yousseria Elsayed Yousef, Essam A. Abo El-Magd, Osama M. El-Asheer, Safaa Kotb

**Affiliations:** ^1^Department of Pediatric Nursing, Faculty of Nursing, Sohag University, Egypt; ^2^ENT, Faculty of Medicine, Aswan University, Egypt; ^3^Pediatrics, Faculty of Medicine, Assiut University, Egypt; ^4^Public Health, Faculty of Nursing, Assiut University, Egypt

## Abstract

*Background*. Chronic suppurative otitis media (CSOM) remains one of the most common childhood chronic infectious diseases worldwide, affecting diverse racial and cultural groups in both developing and industrialized countries.* Aim of the Study*. This study aimed to assess the impact of educational program on the management of children with CSOM.* Subjects and Methods*. An experimental study design was used. This study included 100 children of both sexes of 2 years and less of age with CSOM. Those children were divided into 3 groups: group I: it involved 50 children with CSOM (naive) who received the designed educational program; control group: it involved 50 children who were under the traditional treatment and failed to respond; group II: those children in the control group were given the educational program and followed up in the same way as group I and considered as group II.* Tools of the Study*. Tool I is a structured questionnaire interview sheet for mothers. It consists of four parts: (1) personal and sociodemographic characteristics of child and (2) data about risk factors of otitis media (3) assessment of maternal practice about care of children with suppurative otitis medi (4) diagnostic criteria for suppurative otitis media. Tool II is the educational program: an educational program was developed by the researchers based on the knowledge and practices needs. This study was carried out through a period of 9 months starting from September 2013 to May 2014. The educational program was implemented for mothers of children with CSOM in the form of 5 scheduled sessions at the time of diagnosis, after one week, 1, 3, and 6 months. *Results*. There were significant differences between children who received the educational program and control group regarding the response to treatment after one and 3 months. The percentages of complete cure increased progressively 32%, 60%, and 84% after 1, 3, and 6 months in group I while they were 24%, 44%, and 64% in group II, respectively. Cure (dry perforation) was 64%, 36%, and 12% among children of group I after 1, 3, and 6 months while it was 64%, 44%, and 24% in group II, respectively. The percentages of compliance to the educational program improved with time in both groups: 44%, 64%, and 80% in group I and 32%, 48%, and 56% in group II after 1, 3, and 6 months, respectively. The percentages of cure were statistically significantly higher among children with complete compliance with the educational program in both groups in comparison to those with incomplete compliance (*P* = 0.000 for both). *Conclusions*. From this study we can conclude that the majority of children with CSOM had one or more risk factors for occurrence of the disease; the educational program is effective for management of CSOM (whether cure or complete cure); the higher the compliance of mothers with the program the higher the response rate; regular followup and explanation of the importance of the program played an important role in the compliance with the program.

## 1. Introduction

Chronic suppurative otitis media (CSOM) is defined as a chronic inflammation of the middle ear and mastoid cavity, which presents with recurrent ear discharges or otorrhoea through a tympanic perforation. The disease usually begins in childhood as a spontaneous tympanic perforation due to an acute infection of the middle ear, known as acute otitis media (AOM), or as a sequel of less severe forms of otitis media (e.g., secretory OM). The infection may occur during the first 6 years of a child's life, with a peak around 2 years [1–3]. It is the commonest childhood infectious disease worldwide [4, 5]. The multifactorial nature of otitis media must be stressed. Inadequate antibiotic treatment, frequent upper respiratory tract infections, nasal disease, multiple episodes of AOM, being a member of a large family, and poor living conditions with poor access to medical care are related to the development of CSOM. Poor housing, hygiene, and nutrition are associated with higher prevalence rates. Bottle-feeding, passive exposure to smoking, attendance in congested centers such as day-care facilities, and a family history of otitis media are some of the risk factors for otitis media (Kenna, 1994) [6–9]. The World Health Organization (WHO) global estimate for disabling hearing impairment (degree of severity more than 40 dB) has more than doubled from 120 million people in 1995 to 278 million in 2005. A total of 364 million people have mild hearing impairment, while 624 million are estimated to have some level of hearing impairment and 80% of these live in low- and middle-income countries [10, 11]. Worldwide, there are between 65 and 330 million people affected, of whom 60% receive significant hearing loss. This burden falls disproportionately on children in developing countries [12]. Patients with CSOM respond more frequently to topical therapy than to systemic therapy. Successful topical therapy consists of 3 important components: selection of an appropriate antibiotic drop, regular aggressive aural toilet, and control of granulation tissue. Aural toilet is a critical process in the treatment of CSOM. For the best results, aural toilet should be performed 2-3 times per day just before the administration of topical antimicrobial agents. Failures of topical antimicrobial therapy are almost always failures of delivery [13].

## 2. Aim of the Study

This study aimed to assess the impact of educational program on the management of children with chronic suppurative otitis media.

### 2.1. Research Questions

1. Is there an effect of the designed educational program on the cure of children with CSOM?
2. Is there a difference between the response of naïve children with CSOM who received the program and those who received the program after failure of traditional treatment?

### 2.2. Significance

Worldwide, there are between 65 and 330 million people affected, of whom 60% receive significant hearing loss. This burden falls disproportionately on children in developing countries [12].

## 3. Subjects and Methods

### 3.1. Design

An experimental study design was used in carrying out this study.

### 3.2. Setting

This study was carried out in the outpatient clinics of the Departments of Pediatrics and ENT (Ear, Nose and Throat), Assiut University Hospital.

General objective of this program was to cure the children with chronic suppurative otitis media.

### 3.3. Patients

This study included 100 children of both sexes of 2 years and less of age with chronic suppurative otitis media according to the standard definition. Those children were divided into 3 groups.

1. Group I: it involved 50 children with CSOM (naïve) who received the educational program.
2. Control group: it involved 50 children who were selected from those attending the outpatient clinics under the traditional treatment and failed to respond.
3. Group II: those children in the control group were given the educational program, followed up in the same way as group I, and considered as group II.

The history of the disease, follow-up visits, physician diagnosis, and assessment of the control group were taken from the child's mother and outpatient clinic follow-up card of the child.

### 3.4. Tools of the Study

The following tools were utilized to collect data pertinent to study. These were as follows.

#### 3.4.1. Tool I: A Structured Questionnaire Interview Sheet for Mothers

It consists of four parts:

1. personal and sociodemographic characteristics of child: they include age, sex, residence, birth order, maternal education and occupation, and number of family members;
2. data about risk factors of otitis media: they include history of the disease, presence of dust, sources of vapors, using pacifier, type of feeding, and use of the ideal position for feeding;
3. assessment of maternal practice about care of children with suppurative otitis media: this part was developed by the researcher to evaluate mother's practices given to child with suppurative otitis media;
4. diagnostic criteria for suppurative otitis media: children who were diagnosed with suppurative otitis media were interviewed by the physician to evaluate the case, prescribe the appropriate treatment, and determine the schedule of followup (after one week and 1, 3, and 6 months).

#### 3.4.2. Tool II: The Educational Program

An educational program was developed by the researchers based on the knowledge and practices needs in a form of printed Arabic booklet. It was also supplemented with information based on review of the relevant literature (nursing textbook, journals, internet resources, etc.) about care provided to children with CSOM. This was adopted from [13, 14]. Then, the program was reviewed by a panel of experts before its implementation.

The educational program included the following.

1. Give the antibiotic regularly with the described dose.
2. Clean the external ear from pus with cotton before adding ear drops.
3. Put the ear drops with the correct way.
4. Put a piece of cotton covered with Vaseline before bathing.
5. Separate the infant away from dust, sources of steam and vapors, and those complaining of common cold.
6. Stop using pacifier, sterilize the feeding bottle, and use the ideal position for feeding.

### 3.5. Program Evaluation

Evaluation of the program's success was based on the response of children with CSOM. This evaluation was done after the end of the program and followup (one week, one month, three months, and six months) using definition criteria for response (cure or failure). Complete cure means absence of otorrhoea and spontaneous closure of drum perforation. Cure (dry perforation) means absence of otorrhoea or presence of serous/mucous otorrhoea with negative microbiologic culture or presence of a perforation (hole) in the eardrum without any signs of discharge or fluid behind the eardrum. Failure means presence of purulent or mucopurulent otorrhoea irrespective of culture results or presence of serous/mucous otorrhoea with positive culture [15, 16].

### 3.6. Data Collection

Official approval letters were obtained from the heads of the departments of pediatrics and ENT to conduct the research.

Oral consent was taken from the mother to participate in the study after full explanation of the program and its benefits and hazards and complications of the disease. Those who accepted to participate were interviewed and the program of treatment was clearly explained where they were asked to share actively to achieve the full program. An Arabic translation of all study tools was done and was reviewed by experts in nursing and medicine to ascertain their content validity.

### 3.7. Field of the Work

This study was carried out through a period of 9 months starting from September to May. The educational program was implemented for mothers of children with CSOM in the form of 5 scheduled sessions at the time of diagnosis, after one week, 1, 3, and 6 months. The duration of each session was variable and ranged between 30 and 45 minutes. Each participant obtained a copy of the program booklet that included all the required instructions. The sessions were carried out in the waiting room after the physician observation. At the first visit, clear explanation of the nature of the disease and its possible complications was done by face-to-face interview of mothers of children with CSOM. The detailed items of the designed educational program were explained using a simple Arabic language. The educational program consisted of the following instructions.

1. The antibiotic must be given regularly with the described dose.
2. The mothers were trained to clean the external ear from pus and granulation tissue with pieces of cotton before adding ear drops.
3. They were trained to put ear drops in the correct way in the supine position with the target ear facing the ceiling.
4. Before bathing, a piece of cotton covered with Vaseline must be put in the external ear to keep the ear dry to avoid complications.
5. They were instructed to separate the infant away from dust, sources of steam and vapors, and those complaining of common cold.
6. They were advised to stop using pacifier, sterilize the feeding bottle, and use the ideal position for feeding (upright position when feeding).

In the next visits, the child was evaluated by the physician and clinical diagnosis was established. Then, the mothers were interviewed where the compliance with the items of the educational program was assessed to determine the adherence to the program.

### 3.8. Pilot Study

After developing the tools, a pilot study was implemented for purpose of testing clarity and completeness and to determine the time required for each case. According to the results of pilot, the needed modifications, omissions, and/or additions were done. A jury acceptance of the final forms was secured before actual study work and the reliability was assessed in a pilot study.

### 3.9. Ethical Consideration

An oral consent was taken from all participants in the study. The purpose and nature of the study were explained by the researcher through direct personal communication before starting the study. The data was confidential between mothers and the researchers and was used for the purpose of the research only.

Statistical analysis was done using SPSS-17 statistical software package and Excel for figures. Data were presented using descriptive statistics in the form of frequencies and percentages for qualitative variables and means and standard deviations for quantitative variables. Quantitative continuous data were compared by using Student's *t*-test in case of comparisons between the mean scores of the studied groups. Qualitative studied variables were compared using Chi-square test. Statistical significance was considered at *P* value < 0.05.

## 4. Results

In Table 1, both groups were matched regarding the demographic data except that family members more than 5 were significantly more common among control than study groups (*P* = 0.016).

In Table 2, regarding the possible risk factors for CSOM, children in the control group were significantly more exposed to passive smoking (*P* = 0.035), were more than the 3rd in the birth order (*P* = 0.005), and shared more than 2 children in one room in comparison to those in the study group. More than half of the children with CSOM in the study and control groups were more than the 3rd in the birth order, had more than 2 children in one room, and were exposed to vapors. In addition, more than two-thirds of them were bottle feeders, used pacifier, and were exposed to passive smoking.

In Table 3, when the response to treatment was compared among those who received the educational program (groups I and II) and those who received the traditional treatment (control group), there were significant differences especially after 1 and 3 months. The percentages of complete cure increased progressively, 32%, 60%, and 84% after 1, 3, and 6 months in group I and 24%, 44%, and 64% in group II, respectively. Cure (dry perforation) was 64%, 36%, and 12% among children of group I after 1, 3, and 6 months and 64%, 44%, and 24% in group II, respectively. On the other hand, no evidence of cure was found among control group. After one week of treatment, significant improvement was found among study and control groups in the form of absence of otorrhoea but no significant differences were found among groups. No differences were found between groups I and II regarding response to treatment after 1, 3, and 6 months. The total response to the educational program ranged from 88% in group II to 96% in group I (total response included complete cure and cure).

Table 4 shows that the percentages of compliance with the educational program improved with time in both groups, 44%, 64%, and 80% in group I and 32%, 48%, and 56% in group II after 1, 3, and 6 months, respectively. The percentages of compliance with the educational program were higher among children of group I than group II after 1 and 3 months and the differences were statistically insignificant. After 6 months, the compliance was significantly higher among children of group I than group II (*P* = 0.010). Incomplete compliance after one month among children of group I: 8 (16%) achieved 60%–70% and 18 (36%) achieved 80%–90%. Incomplete compliance after one month among children of group II: 12 (24%) achieved 60%–70% and 6 (12%) achieved 80%–90%. Incomplete compliance after 3 months among children of group I: 14 (28%) achieved 70%–90% while 10 (20%) did in group II. Incomplete compliance after 6 months: 6 (12%) of each group achieved 70%–90%.

Table 5 shows the relation between the compliance with and the response to the educational program among children of both groups after one month. The percentages of cure were statistically significantly higher among children with complete compliance with the educational program in both groups in comparison to those with incomplete compliance (*P* = 0.000 for both).

In Table 6, after 3 months of treatment, the percentages of cure were 100% among those with complete compliance with the educational program among children of both groups. On the other hand, the percentages of cure were 28.6% and 17.6% among children of groups I and II, respectively, who achieved incomplete compliance with the educational program. The differences were statistically highly significant (*P* = 0.000 for both).

In Table 7, after 6 months of treatment, the percentages of cure were 100% among those with complete compliance with the educational program among children of both groups. Although the compliance with the educational program among children of both groups was incomplete, the percentages of cure were 71.4% and 47.1% in groups I and II, respectively. However, the differences were statistically significant (*P* = 0.019 and 0.000, resp.).

## 5. Discussion

Chronic suppurative otitis media (CSOM) is a leading cause of mild to moderate conductive acquired hearing loss worldwide, especially in children, and particularly in developing countries [17–21]. It is characterized by long-standing ear discharge through a persistent perforation of the tympanic membrane. CSOM is believed to develop in early childhood, often following poorly managed acute otitis media, with potential of spilling over into adulthood, accounting for recurrent episodes of chronic discharging ears that can last for many years [22–26].

The current study included 100 children with CSOM. Fifty of them (naïve patients) once diagnosed were selected to receive educational program and followed up for 6 months (group I). The other 50 children were selected from those who received traditional treatment and failed to respond after 3 months (control group). The latter group of children were given the educational program to assess its role after failure of traditional treatment and considered as a new group (group II).

More than half of children with CSOM in the study and control groups were more than the 3rd in the birth order, had more than 2 children in one room, and were exposed to vapors. In addition, more than two-thirds of them were breast and bottle feeders, used pacifier, and were exposed to passive smoking. This agrees with report of van der Veen et al. [27] and Parry et al. [9] who stated that the risk of developing CSOM increases with the following circumstances: multiple episodes of acute otitis media (AOM), living in crowded conditions, being a member of a large family, attending daycare, passive smoking, breast-feeding, socioeconomic status, and the annual number of upper respiratory tract infections.

Children who received the educational program (group I) showed complete cure (this means absence of otorrhoea and spontaneous closure of drum perforation) of 32%, 60%, and 84% after 1, 3, and 6 months in group I and 24%, 44%, and 64% in group II, respectively. The percentages of cure (presence of a perforation in the eardrum without any signs of discharge or fluid behind the eardrum) were 64%, 36%, and 12% among children of group I after 1, 3, and 6 months and 64%, 44%, and 24% in group II, respectively. These results are supported by report of Roland et al. [13] who stated that, among children with CSOM, successful topical therapy consists of 3 important components: selection of an appropriate antibiotic drop, regular aggressive aural toilet, and control of granulation tissue. This is explained by the fact that the external auditory canal and tissues lateral to the infected middle ear are often covered with mucoid exudate or desquamated epithelium. Topically applied preparations cannot penetrate affected tissues until these interposing materials are removed. Failures of topical antimicrobial therapy are almost always failures of delivery. Specifically, failure of delivery describes the inability of an appropriate topical antibiotic to reach the specific site of infection within the middle ear [13]. The educational program included keeping the ear dry during swimming which is in agreement with the reports of Basu et al. [14] and Ball and Bindler [28] that patients with CSOM are usually advised to avoid swimming but if they swim they should put ear plugs and dry their ears afterwards.

After one month, the percentages of complete cure were significantly higher among children with complete compliance with the educational program in both groups in comparison to those with incomplete compliance (*P* = 0.000 for both). After 3 and 6 months of treatment, the percentages of complete cure were 100% among those with complete compliance with the educational program among children of both groups. These results are in agreement with the results of Roland et al. [13] who reported that aural toilet is a critical process in the treatment of CSOM and it must be regular and aggressive. For best results, aural toilet should be performed 2-3 times per day just before the administration of topical antimicrobial agents.

The total response to the educational program ranged from 88% in group II to 96% in group I (total response included complete cure and cure). On the other hand, cure (dry ear) was found in 64% of children of both groups after one month of the program. These results agree with Verhoeff et al. [4, 5] who found that the overall success percentages (dry ear) of topical drops varied from 40% to 100% and concluded that treatment with antibiotic or antiseptic eardrops accompanied by aural toilet was more effective in resolving otorrhoea than no treatment.

## 6. Conclusions

From this study, we can conclude that the majority of children with CSOM had one or more risk factors for occurrence of the disease; the educational program is effective for management of CSOM whether cure or complete cure; the higher the compliance of mothers with the program, the higher the response rate; regular followup and explanation of the importance of the program played an important role in the compliance with the program.

## 7. Recommendations

This program should be applied to a wide scale to validate these results and evaluate its role in the prevention of CSOM especially hearing loss which could not be evaluated in the study.

## Figures and Tables

**Table 1:** Demographic data of study and control groups.

	Group I (*n* = 50)		Control (*n* = 50)		*P* value
	Number	%	Number	%	
Age					0.224
6–12 months	18	36.0	24	48.0
>12 months	32	64.0	26	52.0
Mean ± SD (months)	15.44 ± 6.21		14.16 ± 5.60		0.282
Sex					0.420
Male	26	52.0	30	60.0
Female	24	48.0	20	40.0
Residence					0.532
Rural	34	68.0	31	62.0
Urban	16	32.0	19	38.0
Mother education					0.402
Illiterate	10	20.0	14	28.0
Read and write	6	12.0	2	4.0
Primary	6	12.0	10	20.0
Preparatory	8	16.0	10	20.0
Secondary	10	20.0	8	16.0
University	10	20.0	6	12.0
Mother job					0.629
Housewife	38	76.0	40	80.0
Employer	12	24.0	10	20.0

**Table 2:** Possible risk factors for suppurative otitis media.

	Group I (*n* = 50)		Control (*n* = 50)		*P* value
	Number	%	Number	%	
Family members					0.016^*^
4-5 members	28	56.0	16	32.0
>5 members	22	44.0	34	68.0
Birth order					0.005^*^
2-3	30	60.0	16	32.0
>3	20	40.0	34	68.0
Children in one room					0.043^*^
1-2 children	26	52.0	16	32.0
>2 children	24	48.0	34	68.0
Breast feeding					0.218
Yes	46	92.0	42	84.0
No	4	8.0	8	16.0
Bottle					0.647
Yes	46	92.0	48	96.0
No	4	8.0	2	4.0
Pacifier					0.190
Yes	32	64.0	38	76.0
No	18	36.0	12	24.0
Father smoking					0.349
Yes	36	72.0	40	80.0
No	14	28.0	10	20.0
Baby passive smoking					0.035^*^
Yes	28	56.0	38	76.0
No	22	44.0	12	24.0
Source of vapor					0.102
Yes	26	52.0	34	68.0
No	24	48.0	16	32.0

**Table 3:** Comparison between study and control groups regarding the response to educational program.

	Group I (*n* = 50)		Group II (*n* = 50)		Control (*n* = 50)		*P* value^1^	*P* value^2^	*P* value^3^
	Number	%	Number	%	Number	%			
After 1 month							0.276	0.000^*^	0.000^*^
Complete cure	16	32.0	12	24.0	0	0.0
Cure	32	64.0	32	64.0	0	0.0
No response	2	4.0	6	12.0	50	100.0
After 3 months							0.163	0.000^*^	0.000^*^
Complete cure	30	60.0	22	44.0	0	0.0			
Cure	18	36.0	22	44.0	0	0.0			
No response	2	4.0	6	12.0	50	100.0			
After 6 months							0.069	—	—
Complete cure	42	84.0	32	64.0	—	—			
Cure	6	12.0	12	24.0	—	—			
No response	2	4.0	6	12.0	—	—			

*P* value^1^ between group I and group II, *P* value^2^ between group I and control, and *P* value^3^ between group II and control.

**Table 4:** Comparison between both study groups regarding the compliance with educational program.

	Group I (*n* = 50)		Group II (*n* = 50)		*P* value
	Number	%	Number	%	
After 1 month					0.216
Complete (100%)	22	44.0	16	32.0	
Incomplete (<100%)	28	56.0	34	68.0	
After 3 months					0.107
Complete (100%)	32	64.0	24	48.0	
Incomplete (<100%)	18	36.0	26	52.0	
After 6 months					0.010^*^
Complete (100%)	40	80.0	28	56.0	
Incomplete (<100%)	10	20.0	22	44.0	

**Table 5:** Comparison of the response to and compliance with the educational program between both study groups after one month.

	Physician report	Compliance with the program				*P* value
		Complete		Incomplete		
		Number	%	Number	%	
Group I	Complete cure	14	63.6	2	7.1	0.000^*^
	Cure	8	36.4	24	85.7	
	No response	**0**	**0**	**2**	7.1	
	Total	**22**	**100.0**	**28**	**100.0**	

Group II	Complete cure	12	75.0	0	0.0	0.000^*^
	Cure	4	25.0	30	88.2	
	No response	**0**	**0**	**4**	**11.8**	
	Total	**16**	**100.0**	**34**	**100.0**	

**Table 6:** Comparison between the response to and compliance with the educational program in both study groups after 3 months.

	Physician report	Compliance with the program				*P* value
		Complete		Incomplete		
		Number	%	Number	%	
Group I	Complete cure	22	100.0	6	21.4	0.000^*^
	Cure	0	0.0	20	71.4	
	No response	**0**	**0**	**2**	7.1	
	Total	**22**	**100.0**	**28**	**100.0**	

Group II	Complete cure	16	100.0	6	17.6	0.000^*^
	Cure	0	0.0	22	64.7	
	No response	**0**	**0**	**6**	17.6	
	Total	**16**	**100.0**	**34**	**100.0**	

**Table 7:** Comparison between the response to and compliance with the educational program in both study groups after 6 months.

	Physician report	Compliance with the program				*P* value
		Complete		Incomplete		
		Number	%	Number	%	
Group I	Complete cure	22	100.0	20	71.4	0.019^*^
	Cure	0	0.0	6	21.4	
	No response	**0**	**0**	**2**	7.1	
	Total	**22**	**100.0**	**28**	**100.0**	

Group II	Complete cure	16	100.0	16	47.1	0.000^*^
	Cure	0	0.0	12	35.2	
	No response	**0**	**0**	**6**	17.6	
	Total	**16**	**100.0**	**34**	**100.0**	
